# Longitudinal associations between television in the bedroom and body fatness in a UK cohort study

**DOI:** 10.1038/ijo.2017.129

**Published:** 2017-06-27

**Authors:** A Heilmann, P Rouxel, E Fitzsimons, Y Kelly, R G Watt

**Affiliations:** 1 Department of Epidemiology and Public Health, University College London, London, UK; 2 Eastman Dental Institute, University College London, London, UK; 3 Institute of Education, University College London, London, UK

## Abstract

**Objective:**

To assess longitudinal associations between screen-based media use (television (TV) and computer hours, having a TV in the bedroom) and body fatness among UK children.

**Methods:**

Participants were 12 556 children from the UK Millennium Cohort Study who were followed from age 7 to age 11 years. Associations were assessed between screen-based media use and the following outcomes: body mass index (BMI), fat mass index (FMI), and overweight.

**Results:**

In fully adjusted models, having a bedroom TV at age 7 years was associated with significantly higher BMI and FMI (excess BMI for boys=0.29, 95% confidence interval (CI) 0.06–0.52; excess BMI for girls=0.57, 95% CI 0.31–0.84; excess FMI for boys=0.20, 95% CI 0.04–0.37; excess FMI for girls=0.39, 95% CI 0.21–0.57) and increased risk of being overweight (relative risk (RR) for boys=1.21, 95% CI 1.07–1.36; RR for girls=1.31, 95% CI 1.15–1.48) at age 11 years, compared with having no bedroom TV. Hours spent watching TV or digital versatile disks were associated with increased risk of overweight among girls only. Computer use at age 7 years was not related to later body fatness for either gender.

**Conclusion:**

Having a TV in the child’s bedroom was an independent risk factor for overweight and increased body fatness in this nationally representative sample of UK children. Childhood obesity prevention strategies should consider TVs in children’s bedrooms as a risk factor for obesity.

## Introduction

Screen-based media have a central role in the lives of today’s children. As technology advances, children now have unparalleled access not only to television (TV) screens but also to computers, game consoles and mobile devices.^1^ In the United Kingdom, TV is still the most consumed medium among children aged 5–11 years, with gaming coming second.^2, 3^

At the same time, rising childhood obesity levels are a national and global public health concern.^4^ In 2014/15, a third of 11-year-old children in England were overweight and almost a fifth were obese.^5^ Ironically, while our screens have become flatter, our children have become fatter. Indeed, a relationship between TV viewing and overweight among children and adolescents has been repeatedly reported.^6, 7, 8, 9, 10, 11, 12, 13^ There is some evidence that a TV in the child’s bedroom might exacerbate the problem.^8, 14, 15^ Potential pathways include snacking/eating calorie-dense foods while watching TV,^16, 17, 18, 19^ exposure to food advertising and product placement^16, 17, 20^ and reduced or disrupted sleep.^21, 22, 23, 24, 25^ An association between screen time and reduced physical activity is often hypothesized but empirical evidence has been contradictory.^12, 26, 27, 28^

UK research on screen use and overweight has so far been mainly cross-sectional and has focused on TV viewing as one of the several risk factors for overweight and obesity among young children, with some conflicting results.^29, 30, 31, 32^ Residual confounding and reverse causality remain potential issues in the existing literature, and little is known about the role of computer games. Further, UK studies have so far relied mainly on body mass index (BMI) as the outcome variable, or overweight and obesity derived from BMI. However, using BMI alone is not the best way of assessing adiposity or body fatness, because it does not distinguish between fat mass and fat-free (lean) mass and can therefore be affected by either. The fat mass index (FMI) is a measure of fat mass (FM) adjusted for height (FM divided by height squared) that is not confounded by fat-free mass^33^ and is therefore a valuable additional indicator of body composition. In this study, we examine longitudinal associations between children’s screen based media use at age 7 years and their BMI, FMI and overweight status at age 11 years while adjusting for a wide range of covariates.

## Methods

### Data

We analyzed data from the UK Millennium Cohort Study (MCS). The MCS is a nationally representative, prospective cohort study that follows the lives of children born between September 2000 and January 2002 in the four countries of the United Kingdom. The sample is geographically clustered and stratified to overrepresent economically disadvantaged areas, areas with high proportions of people from ethnic minority backgrounds, and the three smaller countries of the United Kingdom. The MCS is designed and managed by the UK Centre for Longitudinal Studies (CLS) at the UCL Institute of Education. The data sets are fully anonymized and available for academic use from the UK Data Service.

Data are currently available from 5 waves, collected when the children were 9 months, 3 years, 5 years, 7 years and 11 years. The initial MCS sample at wave 1 included 18 552 families, another 692 families joined at wave 2.^34^ At wave 5, 13 287 families participated (response rate 69%).^35^ The MCS survey weights include unit non-response weights to adjust for attrition between waves.

Ethical approval has been granted for each sweep of the MCS. For wave 5, ethical approval was granted by the Yorkshire and Humber REC (Ref: 11/YH/0203).^34^ All participants provided written informed consent.

### Outcome variables: BMI, FMI, and overweight at age 11 years

The outcome of interest was adiposity (body fatness) at age 11 years, measured via three indicators: BMI, FMI, and overweight. Physical measurements of height, weight and body fat were collected by interviewers who underwent formal training and accreditation. A detailed description of the standardized measurement protocols and process of the interviewer accreditation scheme is available for download from the CLS website.^36^ In brief, children’s height was measured using a Leicester Height Measure Stadiometers (Seca, Birmingham, UK), with the child’s head in the Frankfurt Plane, and recorded to the nearest completed millimeter. Children were asked to wear only light clothing and to remove shoes and socks as well as hair accessories or hairstyles that could affect the reading. Weight and body fat were measured using calibrated Tanita BF-522W scales (Tanita UK, Yiewsley, Middlesex, UK) that were placed on a firm, level surface. We used the derived BMI variable calculated by the CLS. To calculate FMI, we used the following formulas: FM=weight times percentage of body fat, divided by 100; FMI=FM divided by height squared. BMI and FMI were used as continuous variables. Overweight was a binary variable (non-overweight including healthy weight plus underweight vs overweight including obese), based on the age and sex-specific International Obesity Task Force criteria.^37^

### Screen-based media use at age 7 years

We measured screen-based media use at wave 4/age 7 years because it was the first wave to include information on whether or not the child had a TV in his/her bedroom. Three variables were used to measure children’s screen-based media use at age 7 years: whether the child had a bedroom TV; hours spent watching TV or digital versatile disks (DVDs; ‘On a normal week day during term time, how many hours does [child] spend watching television, videos or DVDs?’—<1 h, 1–3 h, >3 h); and hours spent playing on the computer (‘On a normal weekday during term time, how many hours does [child] spend using a computer or playing electronic games outside school lessons?’—none, <1 h, ⩾1 h).

### Covariates

The following covariates were included in the fully adjusted models because they are known to be associated with childhood adiposity and might confound the relationship with screen-based media use^29, 38, 39^: child age, child BMI at wave 2/age 3 years, breastfeeding duration (never, <4 months, ⩾4 months); child ethnicity (White, Mixed, Indian, Pakistani, Bangladeshi, Black Caribbean, Black African, other); maternal BMI at wave 2; maternal education at wave 5 (Level 4/5 (degree or higher degree), Level 3 (2+A levels), Level 2 (5 General Certificate of Secondary Education A–C or 1A level), Level 1 (<5 General Certificate of Secondary Education D–E), overseas qualification only, no qualifications); and family income at wave 5 (Organisation for Economic Co-operation and Development equivalized income quintiles, missing data imputed by CLS). Children’s BMI at age 3 years was included to minimize the possibility of reverse causation. We chose the earliest possible indicator of child BMI because screen use starts long before age 7 years, and therefore using BMI/FMI from later waves would likely result in overadjustment. Maternal BMI was used as a proxy measure to capture the overall food environment in the household as well as potential genetic influences. Again we wanted to use a measure from the earliest possible time point. Because at wave 1 maternal BMI was still influenced by the recent pregnancy, we used maternal BMI from wave 2. Breastfeeding duration was included because it follows a strong social gradient and has been linked to a reduced risk of childhood obesity.^40^

As potential mediating variables, we included bedtimes and markers of physical activity at age 7 years. Bedtimes were assessed via the question ‘On weekdays during term time, does [child] go to bed at a regular time? What time is that?’. Children were classified as having no fixed bedtime if the answer to the first question was ‘no, never or almost never’ or ‘sometimes’, whereas for replies of ‘usually’ and ‘always’ bedtimes were coded as follows: 1930 hours or earlier, between 1930 and 2000 hours, between 2000 and 2030 hours, later than 2030 hours. Physical activity outside school lessons was measured using the question ‘How many days a week does [child] usually go to a club or class to do sport or any other physical activity like swimming, gymnastics, football, dancing, etc.?’ Answers were categorized as: ⩾3 days per week, 2 days per week, 1day per week, or less than once a week.

### Study sample

**Figure 1:**
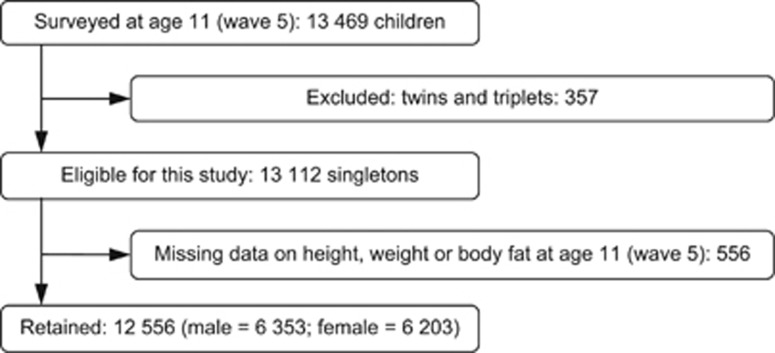
Flow chart of the study participants.

Of the 13 112 children who participated in wave 5 of the MCS and were eligible for this study, 12 556 had complete data on all three outcome measures and were included in the analysis sample. We excluded 357 twins and triplets because the examined outcomes are moderated by multiple gestation pregnancies.^41^ A flow diagram of the study participants is shown in Figure 1.

Only 8147 children had complete data on all covariates. Missingness was highest for maternal BMI at wave 2 (24%). Rates of missingness for all variables are shown in Supplementary Table S1 (online Supplementary Appendix). On average, families of children with missing data were more disadvantaged: among children with missing data, 31% were living in relative poverty (that is, on an income <60% of the median of the population) and 36% of mothers had a university degree, while for those with complete data these were 15% and 45%, respectively. We therefore used multiple imputation by chained equations to handle missing data on covariates due to item non-response.^42^ Imputations were carried out in Stata version 14.1.^43^ The imputation model included all outcome variables, covariates and MCS design variables. The following were included as auxiliary variables: child BMI and FMI at wave 4/age 7 years; maternal education and family income at wave 4; and maternal BMI at wave 5. Preliminary analyses showed that child gender moderated associations between BMI/FMI and the screen time variables (interaction terms statistically significant). Where interactions are present, the stratify-then-impute method is recommended as an ideal solution.^44^ We therefore imputed 25 data sets for boys and girls separately. Results from imputed data were largely similar to those obtained from complete case analyses. We report results from analyses of the imputed data.

### Statistical analyses

All analyses were carried out using Stata version 14.1.^43^ MCS survey weights were employed throughout to account for the stratification and geographical clustering of the data and attrition between the MCS waves. Details on response rates and weighting have been reported elsewhere.^45^

After inspecting crude associations between the three outcome measures and explanatory variables, we estimated a series of regression models. For BMI and FMI, multivariable linear regression was used to estimate associations with screen-based media use while adjusting for potential mediating and confounding factors and BMI at age 3 years. For overweight, we used Poisson regression to estimate relative risk (RR). Poisson or log-binomial regression models are preferred for binary outcomes when the outcome is common (prevalence >10%), because in these cases odds ratios obtained from logistic regression can significantly deviate from risk ratios (but are often interpreted as such) and can therefore be misleading.^46^

We present all results stratified by child gender. For each of the three outcome measures (BMI, FMI and overweight), Model 1 included the three screen time variables and child age only. Model 2 further adjusted for child BMI at age 3 years, breastfeeding duration, child ethnicity, maternal BMI at wave 2 (continuous), maternal education at wave 5 and family income at wave 5. Model 3 was the fully adjusted model, additionally including bedtime and physical activity at age 7 years.

## Results

### Sample characteristics

The analyses included 12 556 singleton children (6353 boys and 6203 girls) with complete information on the three outcome variables. The unweighted mean age of the analysis sample at wave 5 (when the outcomes were measured) was 11.2 years (s.e.=0.33), and 50.6% were boys. The majority of the children were White (84.6%).

Descriptive statistics are shown in Table 1 and Supplementary Tables S2 and S3 (online Supplementary Appendix). All means and proportions shown in descriptive tables are weighted using MCS survey weights. The mean BMI at age 11 years was 19.0 for boys and 19.5 for girls, while mean FMI was 4.1 for boys and 5.0 for girls. About 25% of boys and 30% of girls in the sample were overweight at age 11 years. At age 7 years, 55% of boys and 53% of girls had a TV in their bedroom. Average BMI and FMI, as well as prevalence of overweight, were higher among children who had a TV in the bedroom and among children who spent more time watching TV or DVDs at age 7 years (Table 1).

### Associations between screen-based media use at age 7 years and adiposity at age 11 years

On average, children who had a TV in the bedroom at age 7 years had a significantly higher BMI and FMI at age 11 years compared with those with no bedroom TV (Model 1 in Table 2). Associations appeared to be stronger for girls than for boys. Associations were attenuated but remained statistically significant following adjustment for covariates (Model 3 in Table 2). Excess BMI was 0.29 (95% confidence interval (CI) 0.06–0.52) for boys and 0.57 (95% CI 0.31–0.84) for girls; excess FMI was 0.20 (95% CI 0.04–0.37) for boys and 0.39 (95% CI 0.21–0.57) for girls. Estimates changed very little between Models 2 and 3, suggesting that bedtimes and physical activity did not explain these associations. For boys, hours spent watching TV/DVDs were not associated with BMI and FMI in the adjusted analyses, while for girls, there were statistically significant associations consistent with a dose–response relationship (Models 2 and 3 in Table 2). Again there was only slight attenuation after adjusting for bedtimes and physical activity. Hours spent playing on the computer were not related to BMI or FMI for either gender (Table 2).

Similarly, Poisson regression models (Table 3) showed that the RR of being overweight at age 11 years was higher for children who had a TV in their bedroom at age 7 years, compared with those with no bedroom TV. Again associations were somewhat stronger for girls (RR for boys=1.21, 95% CI 1.07–1.36; RR for girls=1.31, 95% CI 1.15–1.48). After adjusting for covariates, TV/DVD hours at age 7 years were related to an increased risk of overweight at age 11 years only for girls, while there was no association among boys (Models 2 and 3 in Table 3). As before, additional adjustment for bedtimes and physical activity did not substantially affect the results. For both genders, computer hours at age 7 years were unrelated to overweight risk at age 11 years.

## Discussion

Our longitudinal analysis has shown that having a TV in the bedroom is an independent risk factor for increased body fatness in this nationally representative sample of UK children. After extensive adjustment for a wide range of covariates, all three measures of body fatness we assessed were associated with having a TV in the child’s bedroom. Girls who had a TV in their bedroom at age 7 years were at an approximately 30% higher risk of being overweight at age 11 years compared with those who did not have a TV in their bedroom, and for boys the risk was increased by about 20%. The effect size of this increased risk of being overweight is comparable to the risks previously identified for physical inactivity and not having been breastfed.^40, 47^ The number of hours spent watching TV or DVDs was associated with increased body fatness among girls only, showing a dose–response relationship. We found no independent association between hours spent using a computer and body fatness for either gender.

That associations between screen use and overweight/obesity are stronger among girls than among boys has been suggested previously^13, 48, 49^; however, the reasons for these findings remain unclear. Research on the role of children’s computer use in the development of overweight/obesity is still sparse and has produced some conflicting findings.^10, 12, 50^ In our study, computer use was unrelated to later body fatness, which is consistent with the results of one meta-analysis that was, however, conducted >10 years ago.^12^ Further good-quality research is clearly needed.

Our results did not support the notion that screen use affects body fatness via reduced sleep, although this finding should be viewed with a degree of caution. Previous research has shown that screen time affects sleep, and shorter sleep duration has been linked to obesity.^21, 22, 23, 24^ Children’s bedtime, the only measure of sleep available to us, is only an approximation of sleep duration and quality, which might have precluded us from finding evidence for a role of sleep in the association between bedroom TVs and body fatness. In relation to physical activity, the available measures in the MCS are also relatively crude; however, our results are consistent with the findings of three systematic reviews which concluded that sedentary behaviors such as watching TV do not necessarily displace physical activity.^12, 27, 28^

More than half of the 7-year-olds in our sample had a TV in their bedroom. This finding is well in line with other recent UK reports on children’s media use, which also suggest that children increasingly use portable devices, such as tablets and laptops in their bedrooms.^2, 3^

This analysis has some limitations. First, our measures of children’s screen time relied on parental reports. Parents are likely to underestimate the hours children spend using a screen, especially where the child has access to it in their own room. On the other hand, the question whether the child has a TV in their bedroom is straightforward and measurement error for this variable is unlikely to be a concern. Second, owing to the very limited availability of good quality dietary data in the MCS, we were unable to explore whether diet patterns were on the pathway between screen time and body fatness. It is important, however, to acknowledge the strengths of our study. Our longitudinal analysis was conducted on a nationally representative large sample of children from across the United Kingdom, who were followed over a 4-year time period from age 7 to age 11 years. We controlled for a wide range of covariates, including the child’s BMI at age 3 years and maternal BMI, thus minimizing the possibility of reverse causation and accounting for the influence of genetic as well as environmental factors. We measured body fatness using three different indicators: BMI, FMI, and overweight. The inclusion of the FMI improves on previous research that relied mainly on BMI, as FMI represents a more objective index of adiposity that is independent of lean body mass.^33, 51^ Having said that, our findings were consistent across the three outcome measures, and conclusions were very similar irrespective of whether BMI or FMI was used to measure body fatness. Our data therefore provide some evidence that studies using only BMI to assess associations between screen use and body fatness can produce equally informative results.

In conclusion, we have shown that having a TV in a child’s bedroom is a significant independent risk factor for overweight and increased body fatness in a nationally representative sample of UK children. Although our analysis is not causal, our results indicate that future childhood obesity prevention strategies should consider access to TVs in children’s bedrooms as a risk factor for obesity. Future research should investigate potential pathways via sleep duration and diet, examine differences between boys and girls in more depth and utilize interventional designs. Also, further research is needed among older children and adolescents, as the use of screen-based media, including computers, mobile phones and tablets, increases with age.

## Figures and Tables

**Table 1:** Mean BMI, mean FMI and percentage of overweight at age 11 years, by screen-based media use and potential mediators (overall *n*=12 556; boys *n*=6353; girls *n*=6203)^a^

	*% of* n		*Mean BMI (s.e.)*		*Mean FMI (s.e.)*		*% Overweight*	
	*Boys*	*Girls*	*Boys*	*Girls*	*Boys*	*Girls*	*Boys*	*Girls*
Full sample	100.0	100.0	19.03 (0.06)	19.45 (0.06)	4.06 (0.04)	5.04 (0.04)	25.4	29.5
*TV in the bedroom, age 7 years*								
No	44.6	47.2	18.68 (0.08)	18.90 (0.09)	3.85 (0.05)	4.70 (0.06)	21.6	24.3
Yes	55.4	52.8	19.32 (0.09)	19.93 (0.09)	4.23 (0.06)	5.36 (0.06)	28.5	34.2
								
*TV/DVD hours, age 7 years*								
<1 h	18.1	19.8	18.66 (0.12)	18.96 (0.12)	3.79 (0.09)	4.70 (0.08)	21.8	23.7
1–<3 h	64.5	65.7	19.05 (0.07)	19.47 (0.07)	4.09 (0.05)	5.06 (0.05)	25.6	30.2
⩾3 h	17.4	14.5	19.35 (0.15)	19.98 (0.19)	4.25 (0.10)	5.45 (0.13)	28.2	34.7
								
*Computer hours, age 7 years*								
None	10.3	14.0	19.26 (0.20)	19.30 (0.20)	4.07 (2.26)	5.03 (0.14)	26.5	27.2
<1 h	45.5	58.1	18.87 (0.08)	19.34 (0.08)	3.85 (2.21)	4.96 (0.05)	22.8	28.6
⩾1 h	44.2	27.9	19.15 (0.10)	19.74 (0.12)	4.08 (2.49)	5.22 (0.08)	25.4	32.8
								
*Physical activity, age 7 years*								
⩾3 days per week	19.9	18.2	18.69 (0.11)	19.03 (0.12)	3.73 (0.07)	4.72 (0.08)	19.7	26.1
2 days per week	20.3	20.3	18.92 (0.12)	19.24 (0.13)	3.96 (0.08)	4.87 (0.09)	24.9	27.2
1 day per week	25.4	27.0	19.21 (0.13)	19.46 (0.11)	4.16 (0.08)	5.06 (0.07)	26.5	29.6
Less than once a week	34.4	34.4	19.17 (0.11)	19.77 (0.12)	4.24 (0.08)	5.31 (0.08)	28.1	32.7
								
*Bedtime, age 7 years*								
At or before 1930 hours	29.0	30.3	18.66 (0.11)	18.98 (0.11)	3.78 (0.08)	4.73 (0.07)	20.7	24.3
1931–2000 hours	34.8	33.2	18.90 (0.10)	19.38 (0.11)	3.95 (0.06)	4.99 (0.07)	24.0	30.1
2001–2030 hours	15.2	14.6	19.36 (0.15)	19.73 (0.15)	4.22 (0.10)	5.22 (0.11)	28.9	32.5
Later than 2030 hours	10.9	11.4	19.49 (0.17)	20.24 (0.19)	4.52 (0.12)	5.63 (0.14)	31.9	37.3
No regular bedtime	10.0	10.4	19.59 (0.20)	19.74 (0.21)	4.54 (0.14)	5.26 (0.14)	31.6	30.7

Abbreviations: BMI, body mass index; DVD, digital versatile disk; FMI, fat mass index; TV, television.

a Sample sizes unweighted, all results (means and proportions) weighted using the MCS survey weights.

**Table 2:** Multiple linear regression models predicting BMI and FMI at age 11 years, stratified by child gender

	*Estimate (95% CI)*					
	*BMI*			*FMI*		
	*Model 1*	*Model 2*	*Model 3*	*Model 1*	*Model 2*	*Model 3*
*Boys (*n=*6353)*						
TV in the bedroom^a^	0.60 (0.36, 0.84)***	0.33 (0.10, 0.56)**	0.29 (0.06, 0.52)*	0.36 (0.19, 0.53)***	0.23 (0.08, 0.39)**	0.20 (0.04, 0.37)*
						
TV/DVD hours^b^						
1–<3 h	0.32 (0.04, 0.60)*	0.22 (−0.03, 0.47)	0.17 (−0.08, 0.42)	0.25 (0.05, 0.45)*	0.15 (−0.03, 0.33)	0.12 (−0.06, 0.30)
⩾3 h	0.51 (0.09, 0.93)*	0.30 (−0.07, 0.67)	0.22 (−0.15, 0.59)	0.36 (0.09, 0.64)*	0.16 (−0.10, 0.42)	0.10 (−0.16, 0.35)
						
Computer hours^c^						
<1 h	−0.43 (−0.85, −0.02)*	−0.17 (−0.56, 0.22)	−0.19 (−0.58, 0.20)	−0.24 (−0.52, 0.04)	0.00 (−0.24, 0.24)	−0.01 (−0.25, 0.23)
⩾1 h	−0.34 (−0.78, 0.10)	−0.11 (−0.51, 0.29)	−0.15 (−0.55, 0.24)	−0.15 (−0.45, 0.15)	0.07 (−0.18, 0.32)	0.04 (−0.21, 0.28)
						
*Girls (*n=*6203)*						
** **TV in the bedroom^a^	0.94 (0.68, 1.20)***	0.63 (0.36, 0.89)***	0.57 (0.31, 0.84)***	0.61 (0.43, 0.78)***	0.42 (0.24, 0.60)***	0.39 (0.21, 0.57)***
						
TV/DVD hours^b^						
1–<3 h	0.37 (0.10, 0.64)**	0.27 (0.02, 0.52)*	0.23 (−0.03, 0.48)	0.29 (0.10, 0.47)**	0.21 (0.04, 0.38)*	0.18 (0.00, 0.36)*
**⩾**3 h	0.72 (0.27, 1.17)**	0.54 (0.15, 0.94)**	0.45 (0.06, 0.84)*	0.59 (0.28, 0.89)***	0.45 (0.18, 0.72)**	0.39 (0.12, 0.66)**
						
Computer hours^c^						
<1 h	−0.02 (−0.43, 0.39)	−0.05 (−0.43, 0.33)	−0.07 (−0.45, 0.31)	−0.10 (−0.39, 0.19)	−0.09 (−0.35, 0.17)	−0.10 (−0.36, 0.16)
⩾1 h	0.15 (−0.32, 0.61)	0.03 (−0.46, 0.40)	−0.07 (−0.50, 0.35)	−0.00 (−0.32, 0.32)	−0.11 (−0.40, 0.19)	−0.13 (−0.42, 0.16)

Abbreviations: BMI, body mass index; CI, confidence interval; DVD, digital versatile disk; FMI, fat mass index; TV, television. Exposure: screen-based media use at age 7 years. ***P<0.001; **P<0.01; *P<0.05.

Model 1: adjusted for child age at interview.

Model 2: Model 1+child ethnicity, child BMI (wave 2), breastfeeding duration, maternal BMI (wave 2), maternal education (wave 5), and family income (wave 5).

Model 3: Model 2+bedtime (wave 4/age 7 years), physical activity (wave 4/age 7 years).

Weighted results.

a Reference category: no TV in the bedroom.

b Reference category:<1 h during term-time weekday.

c Reference category: none during term-time weekday.

**Table 3:** Poisson regression models predicting overweight at age 11 years, stratified by child gender

	*RR (95% CI)*		
	*Model 1*	*Model 2*	*Model 3*
*Boys (*n=*6353)*			
TV in the bedroom^a^	1.31 (1.16, 1.48)***	1.23 (1.09, 1.39)**	1.21 (1.07, 1.36)**
			
TV/DVD hours^b^			
1–<3 h	1.15 (0.97, 1.35)	1.09 (0.93, 1.27)	1.06 (0.90, 1.23)
⩾3 h	1.22 (0.99, 1.51)	1.11 (0.91, 1.35)	1.06 (0.87, 1.29)
			
Computer hours^c^			
<1 h	0.83 (0.69, 1.00)	0.90 (0.76, 1.08)	0.90 (0.75, 1.07)
⩾1 h	0.86 (0.71, 1.04)	0.92 (0.77, 1.10)	0.91 (0.76, 1.08)
			
*Girls (*n=*6203)*			
TV in the bedroom^a^	1.37 (1.22, 1.53)***	1.33 (1.17, 1.51)***	1.31 (1.15, 1.48)***
			
TV/DVD hours^b^			
1–<3 h	1.22 (1.06, 1.41)**	1.20 (1.04, 1.38)*	1.18 (1.03, 1.36)*
⩾3 h	1.34 (1.13, 1.60)**	1.30 (1.09, 1.55)**	1.27 (1.07, 1.52)**
			
Computer hours^c^			
<1 h	1.03 (0.85, 1.25)	1.03 (0.86, 1.24)	1.03 (0.85, 1.23)
⩾1 h	1.10 (0.90, 1.35)	1.08 (0.89, 1.32)	1.07 (0.88, 1.30)

Abbreviations: CI, confidence interval; DVD, digital versatile disk; RR, relative risk; TV, television. Exposure: screen-based media use at age 7 years. ***P<0.001; **P<0.01; *P<0.05.

Model 1: adjusted for child age at interview

Model 2: Model 1+child ethnicity, child BMI (wave 2), breastfeeding duration, maternal BMI (wave 2), maternal education (wave 5), and family income (wave 5).

Model 3: Model 2+bedtime (wave 4/age 7 years), physical activity (wave 4/age 7 years).

Weighted results.

a Reference category: no TV in the bedroom.

b Reference category:<1 h during term-time weekday.

c Reference category: none during term-time weekday.
