# Supplementary material for: Longitudinal associations between television in the bedroom and body fatness in a UK cohort study
Source: Int J Obes (Lond). 2017 Jun 27;41(10):1503–9. doi: 10.1038/ijo.2017.129 (PMC5630663; doi:10.1038/ijo.2017.129)
Supplement: Supplementary Tables [file ijo2017129x1.docx]

**Supplementary tables for online appendix**

**Table S1** Rates of missingness in covariates (n = 12 556)

|  | n | % |
| --- | --- | --- |
| Bedroom TV, age 7 | 1 170 | 9.3 |
| TV/DVD hours | 1 172 | 9.3 |
| Computer hours | 1 177 | 9.4 |
| Age at wave 5 | 0 | 0.0 |
| Child gender | 0 | 0.0 |
| Child ethnicity | 0 | 0.0 |
| Physical activity, age 7 | 1 169 | 9.3 |
| Bedtime, age 7 | 1 140 | 9.1 |
| Breastfed | 54 | 0.4 |
| Child BMI at wave 2 / age 3 | 1 944 | 15.5 |
| Mother BMI wave 2 | 3 030 | 24.1 |
| Mother NVQ wave 5 | 237 | 1.9 |
| Family income wave 5 | 0 | 0.0 |

**Table S2** Mean BMI, mean FMI and % overweight at age 11, by covariates (n boys = 6 353; n girls = 6 203).

|  | % of n | | Mean BMI (SE) | | Mean FMI (SE) | | % Overweight | |
| --- | --- | --- | --- | --- | --- | --- | --- | --- |
|  | Boys | Girls | Boys | Girls | Boys | Girls | Boys | Girls |
| Full sample | 100 | 100 | 19.03 (0.06) | 19.45 (0.06) | 4.06 (0.04) | 5.04 (0.04) | 25.4 | 29.5 |
| Child age at wave 5 |  |  |  |  |  |  |  |  |
| 10 years | 34.2 | 34.0 | 18.92 (0.10) | 19.21 (0.10) | 4.19 (0.06) | 5.06 (0.07) | 26.6 | 30.2 |
| 11 years | 65.3 | 65.6 | 19.10 (0.06) | 19.55 (0.07) | 4.00 (0.05) | 5.03 (0.05) | 24.7 | 29.0 |
| 12 years | 0.4 | 0.4 | 18.98 (0.53) | 21.97 (0.86) | 3.71 (0.37) | 6.38 (0.65) | 24.6 | 52.6 |
| Child ethnicity |  |  |  |  |  |  |  |  |
| White | 84.3 | 84.8 | 18.95 (0.06) | 19.41 (0.06) | 3.94 (0.04) | 4.98 (0.04) | 23.9 | 29.0 |
| Mixed | 3.5 | 3.3 | 19.50 (0.32) | 19.85 (0.44) | 4.40 (0.23) | 5.38 (0.30) | 32.5 | 33.1 |
| Indian | 2.2 | 1.9 | 19.21 (0.35) | 18.58 (0.32) | 4.78 (0.24) | 4.75 (0.20) | 31.3 | 21.4 |
| Pakistani | 3.5 | 3.8 | 19.39 (0.21) | 19.55 (0.19) | 4.94 (0.17) | 5.36 (0.13) | 33.2 | 33.2 |
| Bangladeshi | 1.4 | 1.5 | 19.49 (0.43) | 19.75 (0.43) | 4.91 (0.34) | 5.56 (0.26) | 34.5 | 34.8 |
| Black Caribbean | 1.3 | 1.1 | 21.20 (0.90) | 20.57 (0.59) | 5.41 (0.86) | 5.69 (0.44) | 38.4 | 39.0 |
| Black African | 2.2 | 1.8 | 19.61 (0.53) | 21.28 (0.60) | 4.67 (0.26) | 6.49 (0.41) | 38.5 | 51.4 |
| Other | 1.7 | 1.9 | 18.70 (0.27) | 18.42 (0.39) | 4.24 (0.22) | 4.70 (0.29) | 24.6 | 20.9 |
| Breastfed |  |  |  |  |  |  |  |  |
| ≥ 4 months | 38.3 | 39.0 | 18.73 (0.09) | 18.97 (0.09) | 4.18 (0.06) | 4.73 (0.06) | 22.7 | 25.4 |
| < 4 months | 31.2 | 30.1 4830.17 | 19.11 (0.10) | 19.50 (0.11) | 4.13 (0.07) | 5.08 (0.07) | 26.1 | 29.8 |
| Never | 30.5 | 30.9 | 19.21 (0.10) | 19.78 (0.10) | 3.85 (0.06) | 5.27 (0.07) | 27.0 | 32.6 |
| BMI wave 2 / age 3 |  |  |  |  |  |  |  |  |
| Healthy weight | 75.7 | 75.9 | 18.32 (0.06) | 18.65 (0.06) | 3.63 (0.04) | 4.52 (0.04) | 18.8 | 21.4 |
| Overweight | 18.2 | 18.5 | 20.62 (0.14) | 21.25 (0.14) | 4.93 (0.10) | 6.18 (0.10) | 40.7 | 49.8 |
| Obese | 6.1 | 5.6 | 23.19 (0.40) | 24.26 (0.26) | 6.83 (0.32) | 8.39 (0.26) | 61.8 | 72.5 |
| Mother BMI wave 2 |  |  |  |  |  |  |  |  |
| Healthy weight | 58.7 | 57.8 | 18.33 (0.06) | 18.61 (0.07) | 3.58 (0.04) | 4.47 (0.04) | 17.7 | 21.0 |
| Overweight | 27.1 | 27.2 | 19.57 (0.12) | 20.01 (0.12) | 4.43 (0.08) | 5.44 (0.08) | 30.9 | 35.7 |
| Obese | 14.3 | 15.0 | 20.91 (0.19) | 21.62 (0.19) | 5.33 (0.15) | 6.53 (0.13) | 46.5 | 51.4 |
| Mother NVQ wave 5 |  |  |  |  |  |  |  |  |
| Level 5 | 7.6 | 8.0 | 18.76 (0.17) | 18.82 (0.15) | 3.81 (0.11) | 4.58 (0.10) | 21.5 | 21.1 |
| Level 4 | 29.4 | 29.3 | 18.61 (0.09) | 19.20 (0.10) | 3.73 (0.06) | 4.84 (0.07) | 19.6 | 27.4 |
| Level 3 | 15.3 | 14.9 | 19.18 (0.15) | 19.35 (0.14) | 4.12 (0.10) | 4.98 (0.10) | 26.4 | 29.3 |
| Level 2 | 25.9 | 25.5 | 19.31 (0.12) | 19.72 (0.11) | 4.25 (0.09) | 5.22 (0.08) | 29.3 | 33.3 |
| Level 1 | 7.6 | 7.3 | 18.85 (0.19) | 19.85 (0.24) | 3.97 (0.13) | 5.35 (0.17) | 25.2 | 33.2 |
| Overseas only | 3.1 | 3.4 | 19.38 (0.34) | 19.10 (0.35) | 4.46 (0.24) | 4.92 (0.24) | 32.4 | 25.0 |
| None | 11.2 | 11.7 | 19.50 (0.23) | 19.86 (0.21) | 4.53 (0.15) | 5.42 (0.15) | 30.8 | 32.0 |
| Family income wave 5 |  |  |  |  |  |  |  |  |
| Richest | 18.4 | 19.1 | 18.50 (0.10) | 18.79 (0.12) | 3.62 (0.07) | 4.57 (0.08) | 18.5 | 23.5 |
| 2^nd^ quintile | 19.5 | 18.6 | 18.72 (0.12) | 19.18 (0.12) | 3.79 (0.06) | 4.83 (0.08) | 22.5 | 27.3 |
| 3^rd^ quintile | 19.8 | 19.5 | 19.12 (0.12) | 19.72 (0.14) | 4.13 (0.08) | 5.23 (0.09) | 26.5 | 32.1 |
| 4^th^ quintile | 20.9 | 21.1 | 19.54 (0.14) | 19.98 (0.14) | 4.42 (0.10) | 5.43 (0.09) | 29.5 | 34.6 |
| Poorest | 21.5 | 21.7 | 19.22 (0.12) | 19.48 (0.13) | 4.27 (0.08) | 5.11 (0.09) | 28.9 | 29.5 |

**Table S3** Percentage of children with a TV in the bedroom at age 7, by covariates (n boys = 6 353; n girls = 6 203).

|  | % of n | | % with bedroom TV at age 7 | |
| --- | --- | --- | --- | --- |
|  | Boys | Girls | Boys | Girls |
| Full sample | 100 | 100 | 55.4 | 52.8 |
| Physical activity, age 7 |  |  |  |  |
| 3 days/week or more | 19.9 | 18.2 | 45.2 | 43.2 |
| 2 days/week | 20.3 | 20.3 | 48.9 | 42.9 |
| 1 day/week | 25.4 | 27.0 | 56.4 | 55.0 |
| < once a week | 34.4 | 34.4 | 64.4 | 62.0 |
| Bedtime, age 7 |  |  |  |  |
| At or before 19.30 | 29.0 | 30.3 | 52.1 | 49.8 |
| 19.31 to 20.00 | 34.8 | 33.2 | 53.7 | 49.3 |
| 20.01 to 20.30 | 15.2 | 14.6 | 56.9 | 56.4 |
| Later than 20.30 | 10.9 | 11.4 | 59.4 | 55.7 |
| No regular bedtime | 10.0 | 10.4 | 63.9 | 64.7 |
| Child age at wave 5 |  |  |  |  |
| 10 years | 34.2 | 34.0 | 54.1 | 49.3 |
| 11 years | 65.3 | 65.6 | 56.1 | 54.5 |
| 12 years | 0.4 | 0.4 | 55.4 | 69.6 |
| Child ethnicity |  |  |  |  |
| White | 84.3 | 84.8 | 58.7 | 56.5 |
| Mixed | 3.5 | 3.3 | 62.9 | 49.9 |
| Indian | 2.2 | 1.9 | 26.6 | 20.7 |
| Pakistani | 3.5 | 3.8 | 26.1 | 19.9 |
| Bangladeshi | 1.4 | 1.5 | 8.9 | 10.9 |
| Black Caribbean | 1.3 | 1.1 | 63.3 | 60.1 |
| Black African | 2.2 | 1.8 | 34.4 | 33.8 |
| Other | 1.7 | 1.9 | 30.3 | 36.9 |
| Breastfed |  |  |  |  |
| ≥ 4 months | 38.3 | 39.0 | 33.0 | 29.6 |
| < 4 months | 31.2 | 30.1 4830.17 | 55.5 | 50.8 |
| Never | 30.5 | 30.9 | 73.1 | 72.8 |
| BMI wave 2 / age 3 |  |  |  |  |
| Healthy weight | 75.7 | 75.9 | 54.6 | 51.5 |
| Overweight | 18.2 | 18.5 | 56.5 | 56.5 |
| Obese | 6.1 | 5.6 | 61.3 | 58.9 |
| Mother BMI wave 2 |  |  |  |  |
| Healthy weight | 58.7 | 57.8 | 52.4 | 48.9 |
| Overweight | 27.1 | 27.2 | 57.5 | 56.6 |
| Obese | 14.3 | 15.0 | 63.5 | 61.1 |
| Mother NVQ wave 5 |  |  |  |  |
| Level 5 | 7.6 | 8.0 | 35.5 | 27.2 |
| Level 4 | 29.4 | 29.3 | 39.6 | 38.9 |
| Level 3 | 15.3 | 14.9 | 58.7 | 54.3 |
| Level 2 | 25.9 | 25.5 | 66.4 | 65.5 |
| Level 1 | 7.6 | 7.3 | 74.4 | 72.1 |
| Overseas only | 3.1 | 3.4 | 47.4 | 53.6 |
| None | 11.2 | 11.7 | 69.6 | 63.4 |

**Table S3 (continued)** Percentage of children with a TV in the bedroom at age 7, by covariates (n boys = 6 353; n girls = 6 203).

|  | % of n | | % with bedroom TV at age 7 | |
| --- | --- | --- | --- | --- |
|  | Boys | Girls | Boys | Girls |
| Family income wave 5 |  |  |  |  |
| Richest | 18.4 | 19.1 | 27.8 | 22.3 |
| 2^nd^ quintile | 19.5 | 18.6 | 44.6 | 41.6 |
| 3^rd^ quintile | 19.8 | 19.5 | 59.5 | 63.2 |
| 4^th^ quintile | 20.9 | 21.1 | 71.6 | 70.0 |
| Poorest | 21.5 | 21.7 | 69.1 | 63.2 |
